# Supplementary material for: DMRforPairs: identifying Differentially Methylated Regions between unique samples using array based methylation profiles
Source: BMC Bioinformatics. 2014 May 15;15:141. doi: 10.1186/1471-2105-15-141 (PMC4046028; doi:10.1186/1471-2105-15-141)
Supplement: Additional file 2 — DMRforPairs output for the comparison of A431-MCF7 and NA17018-NA17105. Please start from the HTML files in each folder. Available via the BMC Bioinformatics website. [file 1471-2105-15-141-S2.zip › 1394847754114233_MOESM2_ESM/A431_MCF7/figures/1607.pdf]

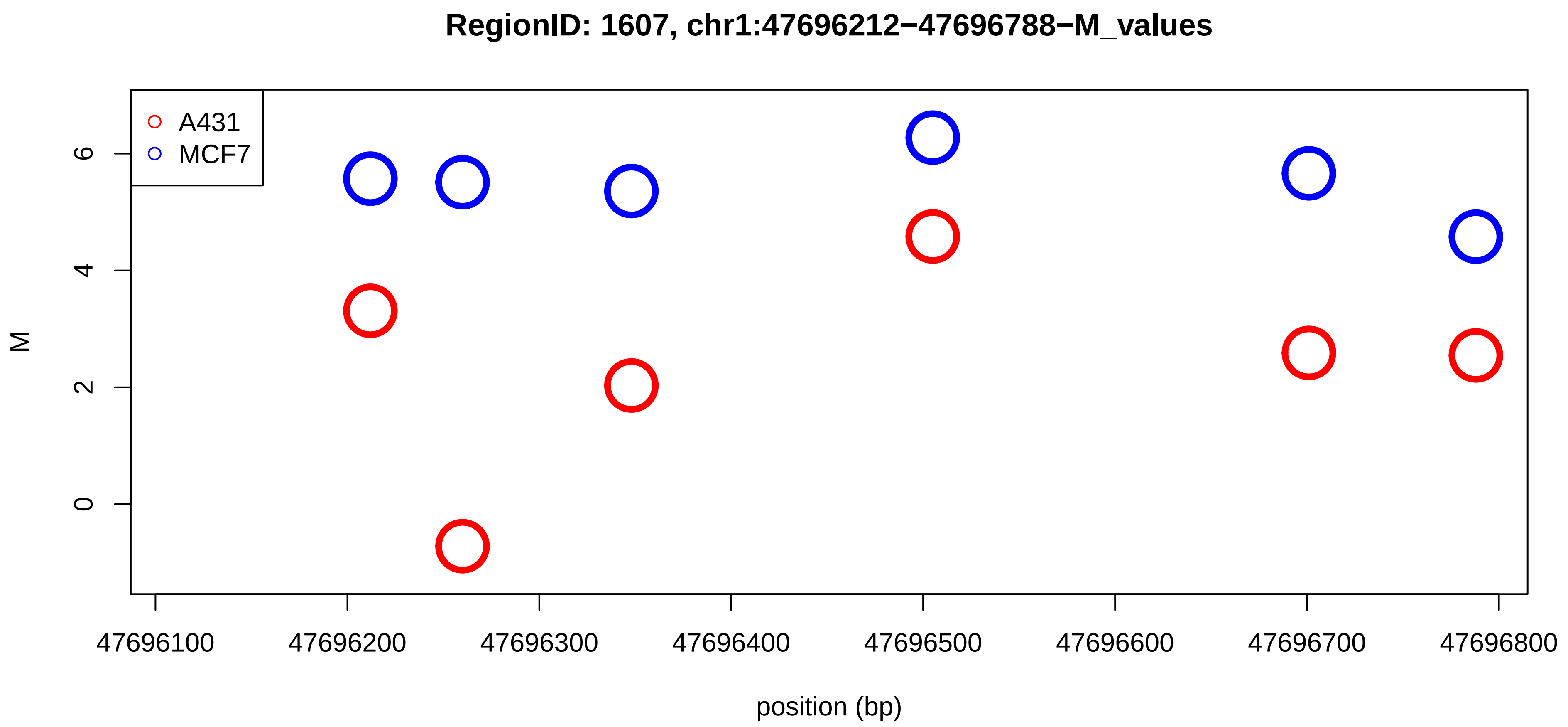
RegionID: 1607, chr1:47696212−47696788−M_values
A431
MCF7
M
0
2
4
6
47696100
47696200
47696300
47696400
47696500
47696600
47696700
47696800
position (bp)

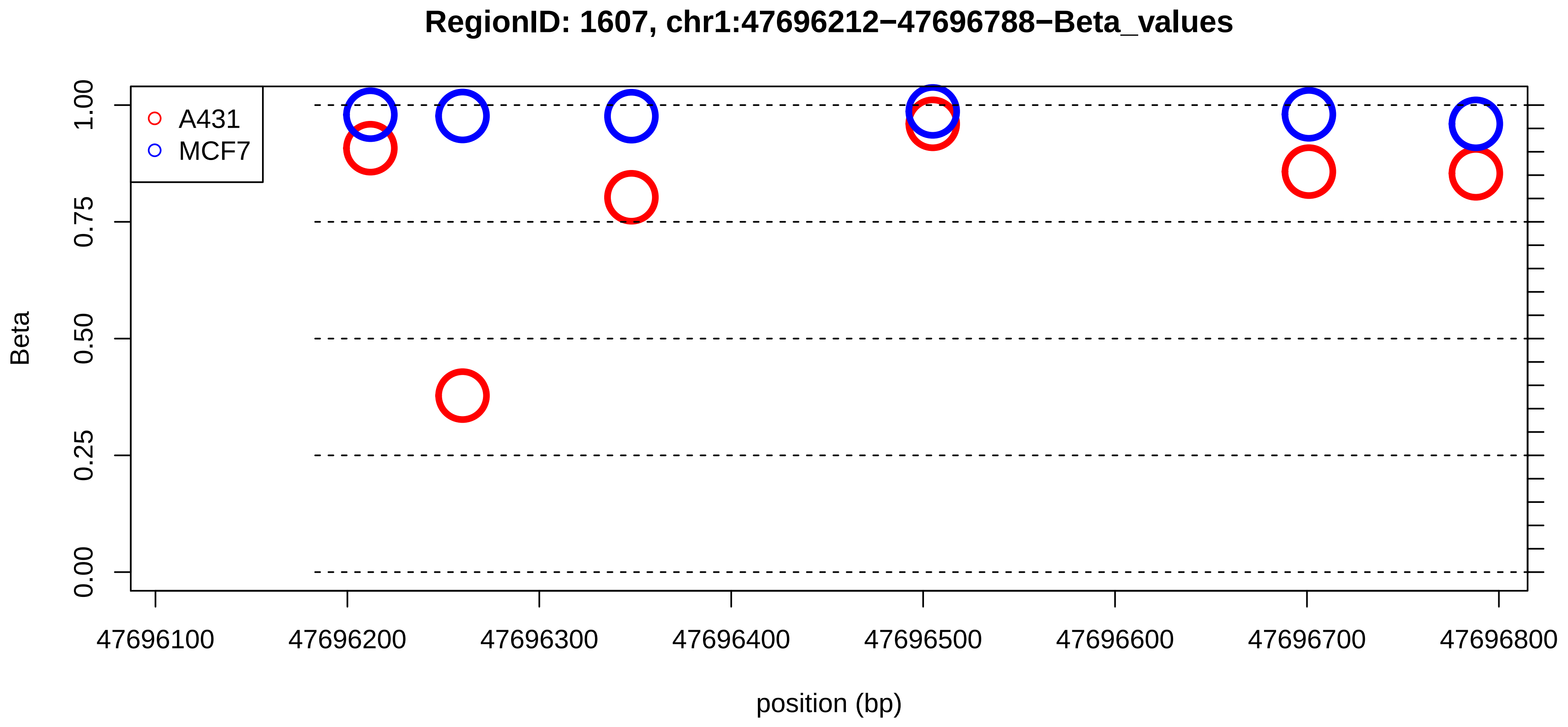
RegionID: 1607, chr1:47696212−47696788−Beta_values
A431
MCF7
Beta
0.00
0.25
0.50
0.75
1.00
47696100
47696200
47696300
47696400
47696500
47696600
47696700
47696800
position (bp)

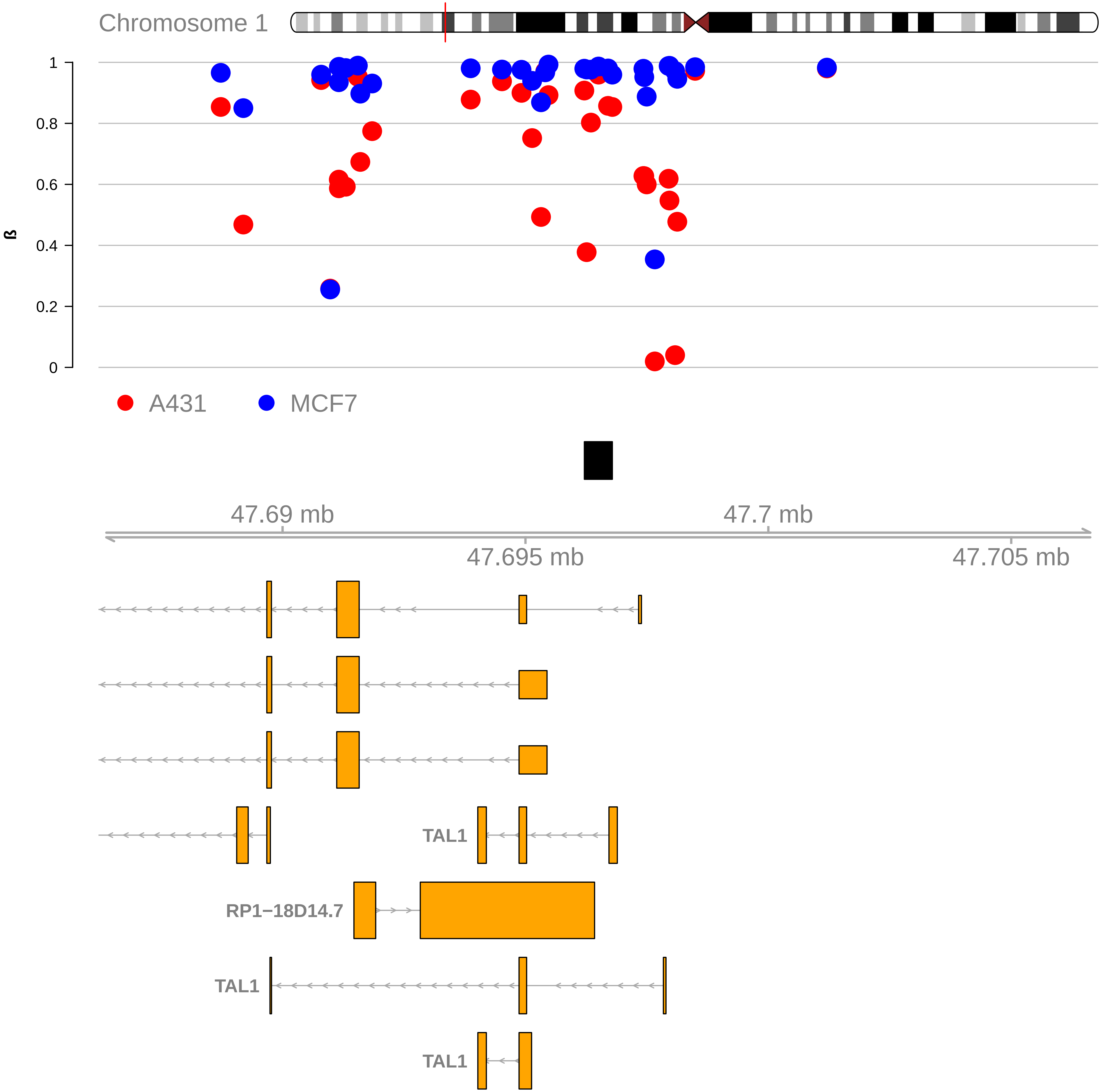

Chromosome 1
ß
1
0.8
0.6
0.4
0.2
0
A431
MCF7
47.69 mb
47.7 mb
47.695 mb
47.705 mb
TAL1
RP1−18D14.7
TAL1
TAL1
